# EN2 as an oncogene promotes tumor progression via regulating CCL20 in colorectal cancer

**DOI:** 10.1038/s41419-020-02804-3

**Published:** 2020-07-30

**Authors:** Yimin Li, Jiaxin Liu, Qing Xiao, Ruotong Tian, Zhengwei Zhou, Yaqi Gan, Yuanyuan Li, Guang Shu, Gang Yin

**Affiliations:** 1 Department of Pathology, Xiangya Hospital, School of Basic Medical Sciences, Central South University, Changsha, China; 2 School of Basic Medical Sciences, Central South University, Changsha, Hunan Province China; 3 China-Africa Research Center of Infectious Diseases, School of Basic Medical Sciences, Central South University, Changsha, Hunan Province China

**Keywords:** Oncogenes, Cell invasion, RNAi

## Abstract

Engrailed-2 (EN2), a member of the engrailed homeobox family, has been shown to be abnormally expressed in a variety of cancers. However, the expression and the clinical significance of EN2 in colorectal cancer (CRC) are largely unknown. Firstly, we found that EN2 acted as an oncogene in CRC. EN2 was upregulated in colorectal cancer tissues compared with adjacent normal tissues. Higher EN2 expression was significantly associated with poorer survival rate. Knockdown of EN2 markedly inhibited proliferation and migration capacities of SW480 cells in vitro, and suppressed tumorigenicity in vivo. Mechanistically, Chemokine ligand 20 (CCL20), a member of the C-C motif chemokine subfamily, was identified as a direct target gene of EN2 in CRC. CCL20 expression was positively correlated with EN2 expression in CRC tissues. Moreover, EN2 promoted the proliferation and migration of CRC cells by regulating the expression of CCL20 in vitro. These results suggest that EN2 plays a critical role in the CRC tumor progression and may serve as a potential target for CRC prevention and therapy.

## Introduction

Colorectal cancer (CRC) is the fourth most common cause for cancer-related death worldwide^1^. Despite improvements in the therapeutic strategies including surgery and chemoradiotherapy, CRC patients still have extremely poor prognosis. Thus, there is an urgent need to identify new functional genes and biomarkers in the pathogenesis of CRC for developing effective treatment strategies.

Homeobox-containing genes are important transcription factors, including HOX, EMX, PAX, engrailed (EN) and so on, which play key roles in both embryonic development and oncogenesis^2,3^. Engrailed, a subgroup of homeodomain-containing family, functions in a variety of animal development processes^4,5^. The result of Bioinformatics analysis implied that EN2, a member of the engrailed homeobox family of homo sapiens, was overexpressed in CRC. Previous research has shown the abnormal expression of EN2 in a wide variety of tumor. For instance, Gómez–Gómez E et al. demonstrated that EN2 was overexpressed in prostate cancer tissues. The treatment of EN2 increased cell proliferation, migration, and PSA secretion^6^. EN2 was also discovered to be expressed ectopically in human breast cancer tissues and cell lines and to promote the adenocarcinoma formation^7^. A recent study reported that EN2 was elevated in serous ovarian tumors compared with the normal ovary^8^. However, the expression and the clinical significance of EN2 in CRC remain elusive so far.

CCL20 plays a crucial role in colorectal cancer and thyroid cancer^9–11^. A large number of literatures provided evidence that CCL20 was significantly increased in CRC^12^. CCL20 regulated CRC proliferation and metastasis by resulting in phosphorylation of p130cas and stimulating ERK-MAP kinase and Akt pathways^13,14^. The regulatory mechanism of CCL20 expression in CRC is not clearly understood. In the present study, we tested the expression of EN2 in colorectal cancer and paired adjacent normal tissues, and then discovered that EN2 was upregulated in the CRC. Moreover, we showed the strong correlation between the high expression of EN2 and the poor survival rate. Through in vitro and in vivo assays, we demonstrated that EN2 significantly enhanced the proliferation and migration of CRC cells by regulating the expression of CCL20.

## Materials and methods

### Bioinformatics analysis

The CRC cohorts, GSE9348^15^, was downloaded from the Gene Expression Omnibus (GEO) database (http://www.ncbi.nlm.nih.gov/geo/). Quantile normalization and log2 transformation were employed for the expression profiles with R software (http://www.bioconductor.org/). GSE9348 has 70 colorectal cancer samples and 12 normal colorectal samples, which were used to detect the expression of EN1 and EN2 in CRC. Next, we verified the expression of EN2 in colorectal tissues in GTEx (507 normal tissue samples) and TCGA databases (568 tumor samples and 44 normal tissue samples). The expression of EN2 was evaluated by means and standard error of the mean (SEM) with Graphpad Prism Software 8.0. The power of EN2 to differentiate between colorectal cancer and normal tissues was evaluated according to ROC curves. Co-expression gene screening for EN2 in CRC patients was performed by cor function in the R platform. The screening criteria were as follows: *P* < 0.05, and | Pearson correlation coefficient | ≥ 0.3. To determine how EN2 affected the prognosis of CRC patients, we performed Kyoto Encyclopedia of Genes and Genomes (KEGG) analysis of EN2 co-expressed genes. These gene functional enrichment analyses were performed using the DAVID (https://david.ncifcrf.gov/).

### Cell lines and cell culture

HEK-293T and CRC cell lines (SW480 and SW620) were purchased from American Type Culture Collection (ATCC; http://www.atcc.org/). HCT8 cell lines were friendly provided by Professor Wancai Yang (Institute of Precision Medicine, Jining Medical University). The normal colon epithelial cell line 8401 were kindly provided by Professor Lunquan Sun (Xiangya Hospital, Central South University). All cell lines were cultured by RPMI-1640 (Biological industries, Kibbutz belt haemek, Israel) with 10% fetal bovine serum (FBS; Biological industries, Kibbutz belt haemek, Israel) at 37 °C with 5% CO_2_.

### Patients and samples

The CRC samples and paired normal tissues were obtained from Xiangya Hospital of Central South University. The patients were informed and signed the informed consent. This work was approved by the Ethics Committee of Xiangya Hospital. The clinical pathological parameters of CRC patients were shown in Table 1.

**Table 1:** Correlations between EN2 expression and clinicopathologic features in 165 colorectal cancer patients.

Clinicopathological feature		Expression of EN2			
	Total (165)	Low (*n* = 64)	High (*n* = 101)	*χ*^2^	*P*-value
Age (years)				0.984	0.321
≤65	90	38	52		
>65	75	26	49		
Gender				2.457	0.117
Male	96	32	64		
Female	69	32	37		
Tumor location				0.011	0.915
Colon	123	48	75		
Rctum	42	16	26		
Tumor size				10.531	**0.001**
≤5 cm	77	40	37		
>5 cm	88	24	64		
Histology grade				9.726	**0.002**
Well	78	40	38		
Moderate/poor	87	24	63		
TNM stage				19.498	**<0.001**
I/II	61	37	24		
III/IV	104	27	77		

The bold number represents the *P*-values with significant differences.

### Plasmids, transfection, and infection

For overexpression of EN2 in CRC cells, the full-length EN2 cDNA was amplified from SW480 cell and then inserted into pcDNA 3.1 vector. The promoter regions of CCL20 and the corresponding mutant were cloned into the pGL3-basic vector. The PLKO.1 vector was used to clone the shRNAs targeting EN2. The pcDNA 3.1-EN2-Flag and pCDH-CMV-MCS-EF1-CCL20 plasmids were obtained from CUSABIO (Wuhan, China). To silence EN2, two small interfering RNAs (siRNAs) were purchased from RiboBio (Guangzhou, China). The siRNAs sequences targeting EN2 were as follows: EN2 si#1: AGTTCCAGACCAACAGGTA; EN2 si#2: ACCCGAACAAAGAGGACAA. Transient transfection of siRNA or plasmid was performed by using a standard protocol from the jetPRIME DNA & siRNA Transfection Reagent (PolyPlus-transfection, France). The transfected cells were harvested after 48 h. The overexpression and the silence efficiency were determined by Western blot. Stable cell lines expressing EN2 shRNA were generated via retroviral infection. Briefly, independent shRNAs against EN2 were constructed using a pLKO.1 vector. The HEK-293T cells were transfected with PLKO.1 based shRNAs, pREV, pGag, and pVSVG at the ratio of 2:2:2:1. The virus particles were collected 48 h after transfection. The SW480 cells were infected with recombinant lentivirus transducing units using 1 μg/ml polybrene (Sigma–Aldrich, St. Louis, MO), and the stable cell lines were selected for 14 days with 1 μg/ml puromycin.

### Cell proliferation assays

Cell proliferation was tested by Cell Counting Kit-8 (CCK8) assay, Colony formation assay and EDU assay. CCK8 assay was carried out as previously described^16^. For colony formation assay, 300 cells were seeded in 12-well plates for 10 days. Cell colony was fixed and stained with crystal violet and the numbers of colonies were estimated using the ImageJ. EdU (RiboBio, Guangzhou, China) assay was performed according to a standard protocol as described. All the experiments were repeated at least three times.

### Cell migration assays

The migration ability of the cells was determined by transwell assay and wound healing assay, which were carried out as previously described^16^. For transwell assay, a total of 1 × 10^6^ transfected cells were seeded into the top chamber of a 24-well polycarbonate transwell filter (8 µm pore size, Corning Incorporated, USA). Media containing 20% FBS was placed in the lower chamber. After incubation for 12 h, the migration cells on the lower surface were fixed with 4% paraformaldehyde and stained with 5% crystal violet. The numbers of crystal violet stained cells in five random fields were counted using an inverted microscope (Olympus, Tokyo, Japan). For wound healing assay, cells were seeded in 6-well plates, and grown to 100% confluency. A 10-µl pipette tip was applied to wound the cell monolayer. Subsequently, the cells were incubated in free-serum medium and cultured for 48 h. Wound closure was photographed using an inverted microscope.

### RNA extraction and real-time PCR

All RNA was extracted with Trizol reagent (Vazyme, Nanjing,China), and the first-strand cDNA was inversely transcribed from total RNA (2 μg) using GoScript Reverse Transcription System (Promega, Madison, WI, USA). Then, qRT-PCR was performed to analyze cDNA using GoTaq qPCR Master Mix (Promega, Madison, WI, USA) on an ABI Prism 700 thermal cycler (Applied Biosystems, Foster City, CA, USA). Specific steps for qRT-PCR have been shown in our previous studies^16^. Ninety-six-well PCR Plates were used for all tests (NEST Biotechnology, No.402301, China). The qRT-PCR conditions for glyceraldehyde-3-phosphate dehydrogenase (GAPDH), EN2, and CCL20 were 95 °C 10 min, followed by 40 cycles of 95 °C 15 s, 60 °C 60 s. All experiments were carried out in triplicate, and the relative expression of interest genes was normalized to the expression of GAPDH, using 2^‒ΔΔct^ method. Primer sequences were as follows: EN2 (forward primer: AGGAGCTGAGCCTCAACGAGTC; reserve primer: CTTGGCTGTGGTGGAGTGGTTG); CCL20 (forward primer: TGCTGTACCAAGAGTTTGCTC; reserve primer: CGCACACAGACAACTTTTTCTTT); GAPDH (forward primer: CTGGGCTACACTGAGCACC; reserve primer: AAGTGGTCGTTGAGGGCAATG).

### Western blot

Total protein was extracted with RIPA buffer containing protease inhibitors and the concentration was measured with the BCA kit. SDS-PAGE was performed with 30 μg total protein. The methods can be found in early study^16^. Primary antibodies included anti-EN2 (1:500, ab45867, abcam, UK), anti-Lamin B1 (1:1000, 12987-1-AP, proteintech, China), and anti-GAPDH (1:5000, 60004-1-Ig, proteintech, China).

### Enzyme-linked immunosorbent assay (ELISA)

The supernatant of treated cells was collected and stored at −80 °C. CCL20 cytokine levels were measured through the highly sensitive ELISA kits (4 A biotech, CHE0061, China). The follow-up steps were carried out according to manufacturer’s instructions.

### Nuclear and cytoplasmic fractionation assay

Nuclear and cytosolic protein were extracted by Nuclear and Cytoplasmic Protein Extraction Kit (Beyotime, P0028, China). The follow-up steps were carried out according to manufacturer’s instructions. For the cytoplasmic extract, SW480 cells were lysed in P0028-1 and P0028-2 buffer. Nuclei were then resuspended in P0028-3 buffer.

### Immunohistochemical staining

The protein expression of EN2 and CCL20 were determined by immunohistochemical (IHC) carried out as previously described^16^. The primary antibodies were as follows: anti-EN2 (1:100, MAB2600, USA), anti-CCL20 (1:50, CSB-PA389153, China). Five areas of positive stains were selected to estimate at high (×200) magnification using light microscopy by two pathologists who were blinded to the clinicopathological data. The staining index (SI) was calculated as tissue staining intensity and percentage. The percentage of cells was graded as follows: 1 (0–25%), 2 (26–50%), 3 (51–75%), or 4 (>75%). Staining intensity was scored as follows: 0 (negative), 1 (weak), 2 (moderate), and 3 (strong); the percentage of cells was scored in the following four categories: 1 (0–25%), 2 (26–50%), 3 (51–75%), or 4 (>75%). SI = staining percentage × intensity. Samples with SI ≥ 6 were determined as high expression, and those with SI < 6 were determined as low expression.

### Luciferase reporter assay

HEK-293T, SW480, and HCT8 cells were inoculated into a 24-well plate with a density of 1 × 10^5^cell/ml, and when the confluence reached 70%, the plasmids were con-transfected with the jetPRIME^®^ DNA & siRNA Transfection Reagent (PolyPlus-transfection, France). After 48 h, the cells were lysis and measured by Dual–Glo luciferase assay (Promega, Madison, WI, USA) and the luciferase activity was obtained by normalizing with Renilla for each sample. Each experiment was repeated three times.

### Chromatin immunoprecipitation (ChIP)

ChIP assay was performed using ChIP kit (Santa Cruz Biotechnology, CA, USA) according to the manufacturer’s instructions. First, EN2-Flag was overexpressed in HCT8 cells. Cells were crosslinked in 1% formaldehyde for 10 min at 37 °C and then lysed in SDS buffer. Sonication was used to fragment the DNA. ChIP for EN2 was performed using a Flag antibody (CST, #14793 S). Eluted DNA fragments were analyzed by qPCR using the specific primers are listed as follows: CCL20-P1 (forward primer: AGTCCTTCGATGCCTGCTAAG; reserve primer: ACCGTGCCCAGCAGAATAG), CCL20-P2 (forward primer: CTATTCTGCTGGGCACGGT; reserve primer: GCCTCAGCCTCCTGAGTAG), CCL20-P3 (forward primer: GTCTGATATAGGCATCACCAACTCC; reserve primer: CCTATCAGCAGTAGCTAGTCAGC).

### Animal study

All animal protocols were approved by the Institutional Animal Care and Use Committee of Central South University (Changsha, China) as previously described^16^. Stable cells (3 × 10^6^) of EN2 knocked down were subcutaneously injected into the upper limb flank of 5-week-old BALB/c (nu/nu) nude mice (*n* = 5/group). The tumor size was measured and recorded. All tumor grafts were excised, weighed, fixed in formalin and embedded in paraffin, of which sections were stained with HE and IHC.

### Statistical analysis

All quantitative data were presented as the mean ± standard error of the mean (SEM) deviation from at least three independent experiments. Statistical analysis was carried out using SPSS version 20.0 and Graphpad Prism Software 8.0. The significance of difference between groups was analyzed by Student’s *t*-test. Survival curves were analyzed by the Kaplan–Meier analysis with log-rank test. Receiver operating characteristic (ROC) curve analysis was done by RStudio. *P*-value of less than 0.05 considered to be significant.

## Results

### EN2 is upregulated in colorectal cancer and related to poor prognosis of patients

**Fig. 1:**
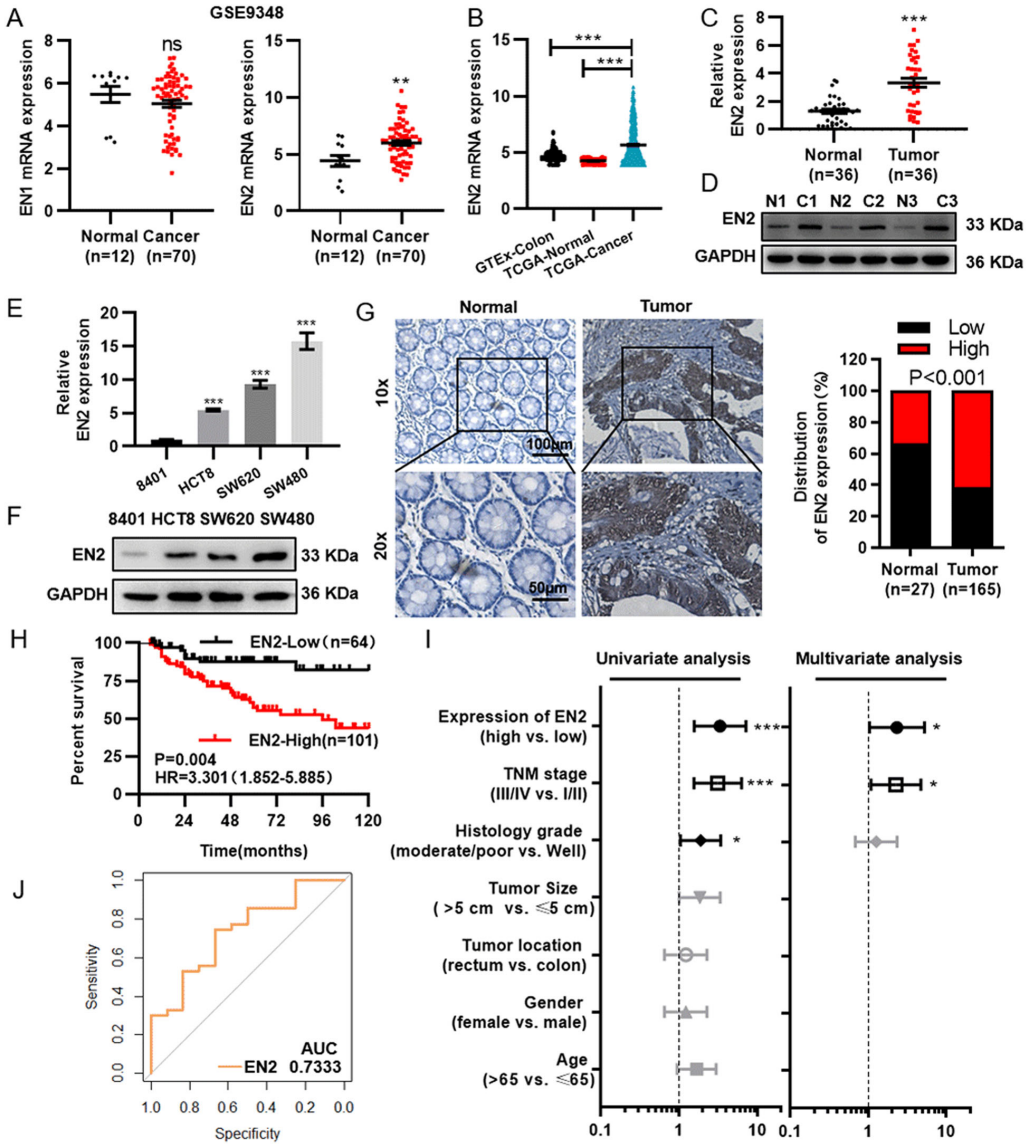
Elevated EN2 expression is associated with colorectal cancer progression. **a** The expression of EN1 (left) and EN2 (right) in CRC samples in GSE9348 dataset. **b** The expression of EN2 in CRC samples in GTEx and TCGA dataset. **c**, **d** The expression of EN2 analyzed by qRT-PCR (**b**) and western blotting (**c**) in CRC samples and adjacent normal colorectal samples. **e**, **f** The expression of EN2 analyzed by qRT-PCR (**d**) and western blotting (**e**) in 8401 and CRC cell lines. **g** The protein expression of EN2 detected by immunohistochemistry (IHC) in CRC and adjacent normal colorectal samples. Right is the quantification data for IHC. Top: original magnification, ×100. Scale bar: 100 µm; Bottom: original magnification, ×200. Scale bar: 50 µm. **h** Kaplan–Meier analysis of the overall survival rate of 165 CRC patients with low or high expression level of EN2. **i** Univariate and multivariate Cox regression analysis of different prognostic factors in CRC patients. **j** Receiver operating characteristic (ROC) curves for detecting CRC patients in GSE9348. Data are presented as mean ± SEM from three independent experiments. *P < 0.05, **P < 0.01, ***P < 0.001, ns no significance.

The homo sapiens Engrailed-1 (EN1) and Engrailed-2 (EN2) genes encode homeobox-containing transcription factors that are the homologs of engrailed gene. To identify critical engrailed genes that contribute to the colorectal cancer tumorigenesis, we analyzed the mRNA expression of EN1 and EN2 in GSE9348, in which EN2 was significantly upregulated in CRC (Fig. 1a). Next, we would like to validate whether the expression of EN2 from GSE9348 is consistent with the expression in colorectal tissues from TCGA and GTEx. As expected, the results were so (Fig. 1b). Furthermore, we verified the expression of EN2 in CRC tissues and cell lines. The expression of EN2 was examined by qRT-PCR and Western blot in the colorectal cancer tissues and adjacent normal tissues, together with the CRC cell lines (HCT8, SW620, and SW480) and human intestinal epithelial cell line (8401). As is shown in Fig. 1c, d, both the protein and mRNA levels of EN2 were significantly upregulated in colorectal cancer tissues. All CRC cell lines showed significant high expressions of EN2 compared to 8401 cells (Fig. 1e, f).

To make a further assessment of the correlation between the expression level of EN2 and the clinicopathological features of CRC, IHC was used to detect the protein level of EN2 in paraffin embedded tissues. The results showed that EN2 expression was significantly higher in tumor tissues compared to the normal tissues (Fig. 1g). The correlation between EN2 protein and the clinicopathological characteristics of patients was observed, which revealed that the expression level of EN2 was significantly associated with the tumor size, histological grade and TNM stage (Table 1). Further, with the aim of exploring whether EN2 could be a potential prognostic factor for colorectal cancer, we performed Kaplan–Meier survival analysis on the basis of the EN2 expression levels of 165 colorectal cancer patients. The results suggested that the CRC patients with the low EN2 expression had the higher overall survival rate than those with relatively high expression (Log-Rank, *P* < 0.05) (Fig. 1h).

Meanwhile, univariate and multivariate Cox regression analysis was performed on the paraffin embedded tissues to assess the correlation between EN2 and survival in the presence of clinicopathological characteristics. The analysis showed that the expression of EN2 was an independent prognostic factor for patients’ survival, in addition to TNM stage (Fig. 1i). Moreover, receiver operating characteristic (ROC) curve analysis was also performed. We found that the expression of EN2 was enough to distinguish CRC cancer patients’ tissues from normal ones (Fig. 1j).

### EN2 knockdown restrains proliferation and migration of CRC cells in vitro

**Fig. 2:**
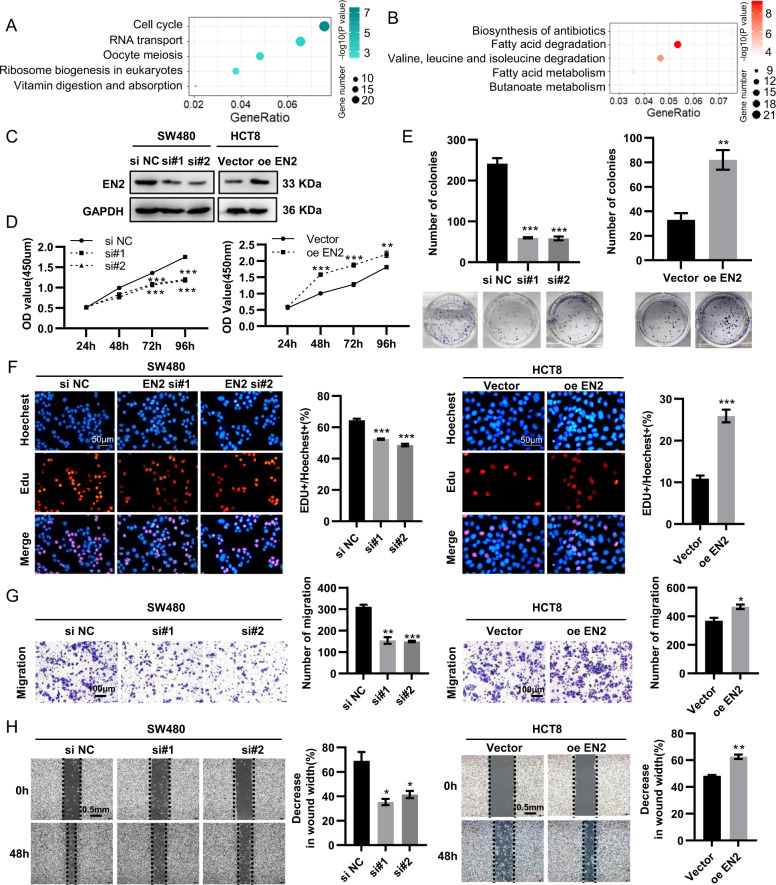
EN2 knockdown restrains the proliferation and migration of CRC cells. **a**, **b** KEGG pathway enrichment analyses of EN2 co-expression genes (**a** displays the genes in positive relation with EN2, while **b** displays those in negative relation). **c** The knockdown of EN2 in SW480 cells and the overexpression of EN2 in HCT8 cells was identified respectively by western blotting. **d**, **f** The capacity of cell proliferation was determined by CCK8 assay (**d**), cell clone-formation assay (**e**), and Edu assay (**f**). Scale bar: 50 µm. **g** The capacity of cell migration was assessed by transwell assay. Scale bar: 100 µm. **h** The capacity of cell migration was assessed by wound healing test. Scale bar: 0.5 mm. Data are presented as mean ± SEM from three independent experiments. *P < 0.05, **P < 0.01, ***P < 0.001.

To further explore the role of EN2 in CRC and the mechanisms of its oncogenic function, we screened the EN2 co-expressed genes from GSE9348 (|R| ≥ 0.3) and then performed KEGG pathway analysis on those genes, among which some positively correlated with EN2 enriched in cell cycle, RNA transport and oocyte meiosis, while others negatively correlated with it enriched in metabolic pathways, biosynthesis of antibiotics and fatty acid degradation (Fig. 2a, b). Therefore, the results from this study indicated that EN2 was related with a range of cancer-associated pathways. Also, the results of IHC revealed that there was the correlation between EN2 and the tumor size, suggesting that EN2 might be involved in the regulation of proliferation (Table 1). Next, we knocked down the EN2 in the SW480 cell line, which had high EN2 expression, whereas overexpressed the EN2 in the HCT8 cell line, which had relative low expression (Fig. 2c). To verify the effect of EN2, we detected the cell proliferation and viability of colorectal cancer cells by CCK8, EDU, and cell clone-formation assay. Further, silencing EN2 in CRC inhabited cell growth distinctly, while overexpressing EN2 promoted the growth (Fig. 2d–f). To further study the effect of EN2 on cell migration, we performed the transwell assay and wound healing test and discovered the silence of EN2 significantly repressed CRC cell migration, whereas the opposite results were observed in CRC cells with the overexpression of EN2 (Fig. 2g, h).

### EN2 knockdown prohibits tumorigenicity in vivo

**Fig. 3:**
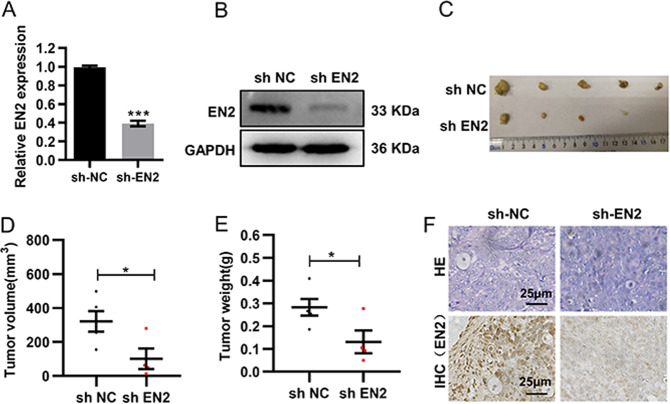
EN2 knockdown restrains tumor growth in vivo. **a**, **b** The knockdown of EN2 in SW480 cells were identified by qRT-PCR (**a**) and western blotting (**b**). **c** The tumor growth curves of shRNA-EN2 and shRNA-NC groups in the xenograft mouse model. **d**, **e** The tumor volume (**d**) and weight (**e**) of nude mice were measured. **f** Representative images of H&E staining and IHC staining in subcutaneous tumors from nude mice. Scale bar: 25 µm. Data are presented as mean ± SEM. *P < 0.05.

To further verify the effect of EN2 on tumorigenesis in vivo, we established the subcutaneous xenograft tumor models using SW480 cells with or without EN2 knockdown. As shown in Fig. 3a, we constructed shRNA vectors of EN2 and transfected them into SW480 cells to establish a cell line expressing low level of EN2 stably (Fig. 3a, b). Then, these cells were subcutaneously implanted into the nude mice, and tumor growth was subsequently quantified. The results revealed that EN2 knockdown significantly reduced the tumor growth (Fig. 3c), as well as resulted in the decrease of both volume and weight of tumors (Fig. 3d, e). Further analysis of xenografted CRC tissues by immunohistochemical staining confirmed that the protein level of EN2 decreased in the EN2 knockdown group (Fig. 3f). Collectively, these results indicated that EN2 played a critical role in CRC cell growth in vivo.

### EN2 is mainly localized in the nucleus as a transcription factor

**Fig. 4:**
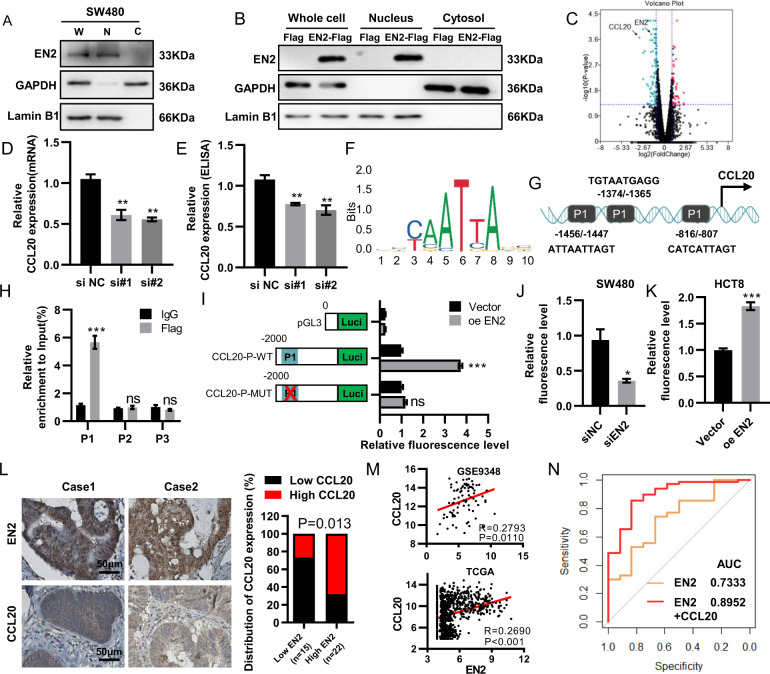
EN2 directly regulates CCL20 expression in CRC cells. **a** Nuclear and cytoplasmic fractionation assay confirmed the nuclear localization of endogenous EN2 in SW480 cells. Nuclear and cytoplasmic extracts were analyzed by western blotting. **b** The localization of EN2-Flag in HCT8 cells was identified by nuclear and cytoplasmic fractionation assay and western blotting. **c** Volcano plot of differentially expressed genes (DEGs) of SW480 cells after EN2 knocked down. The horizontal line at *P*-value = 0.05; vertical line at |log2FC| = 1. **d**, **e** The expression of CCL20 was determined by qRT-PCR (**e**) and ELISA (**f**) after EN2 was knocked down. **f** The EN2 binding motif was predicted from JASPAR matrix models. **g** The schematic structures of EN2 putative binding sites in the CCL20 promoter. **h** ChIP analysis of EN2 binding sites to the CCL20 promoter. **i** The CCL20-P-WT and CCL20-P-MUT luciferase activity in 293 T cells was confirmed by luciferase reporter assay after EN2 was overexpressed. **j**, **k** Promoter luciferase reporter assays of CCL20 in HCT8 cells with EN2 overexpressed (**j**) and in SW480 cells with EN2 knocked down (**k**) were performed. **l** The protein expression of EN2 and CCL20 detected by Immunohistochemistry staining in CRC tissues. Scale bar: 50 µm. **m** The analysis of correlation between EN2 and CCL20 expression levels in CRC tissues in GSE9348 and TCGA database. **n** ROC curves for the diagnosis of CRC patients in GSE9348. Data are presented as mean ± SEM from three independent experiments. *P < 0.05, **P < 0.01, ***P < 0.001.

Previous studies had revealed that EN2 was located in the cytoplasm of breast and prostatic cancer^7,17^. However, our results showed EN2 was mainly located in the nucleus of colorectal cells (Fig. 1f). Nuclear and cytoplasmic fractionation assay confirmed the nuclear distribution of EN2 in SW480 cells (Fig. 4a). Additionally, we also transfected EN2-Flag into HCT8 cells (Fig. 4b). Western blot further confirmed the nuclear distribution of EN2-Flag. Combined, the results raised the conjecture that EN2 might be a transcription factor involving in the progression of colorectal cancer.

To investigate the molecular mechanism of EN2 in colorectal cancer, we explored the gene expression profiles change of CRC cells after knocking down EN2 through RNA sequencing (Fig. 4c). Gene ontology (GO) and KEGG pathway enrichment analyses were applied to analysis the differently expressed genes (DEGs). In the biological process group, DEGs were mainly enriched in cell response to lipopolysaccharide, wound healing and neutrophil chemotaxis. Cell component analysis revealed that DEGs were mainly gathered at the cell surface, extracellular region, and intracellular. In addition, the molecular function analysis showed that DEGs were mainly enriched in the sequence-specific DNA binding, the growth factor activity and the cytokine activity (Supplementary Fig. 1A). KEGG pathway showed that DEG gathered at TNF pathway, cytokine–cytokine receptor, HTLV-1 signing pathway and so on (Supplementary Fig. 1B).

### CCL20 acts as the target gene of EN2

Among these DEGs, CCL20 expression exhibited the highest fold change in si-EN2 group and thus CCL20 was selected for further experimental validation (Fig. 4c). The knockdown of EN2 notably suppressed the expression of CCL20, which was determined by qRT-PCR and ELISA (Fig. 4d, e). To confirm the regulatory relationship between EN2 and CCL20 in CRC, we performed JASPAR database analysis and discovered that the promoter of CCL20 gene contained the EN2 binding sites (Fig. 4f). Next, we identified the binding sites of EN2 on the CCL20 promoter using ChIP assay. Three pairs of primers according to the predicted binding sites were used for qPCR after ChIP (Fig. 4g). ChIP assay demonstrated that EN2 interacted physiologically with the P1 (−1456 to −1447 bp) region on the promoter of the CCL20 (Fig. 4h). Furthermore, we generated luciferase reporter plasmids: CCL20-P-WT, containing −2000 bp upstream of the transcriptional start site, and CCL20-P-MUT, containing a mutated in P1 (−1456 to −1447 bp) region (Fig. 4i). Luciferase reporter assay confirmed that EN2 prominently increased luciferase activity in CCL20-P-WT group not in CCL20-P-MUT in 293 T cells (Fig. 4i). Furthermore, the overexpression of EN2 increased the CCL20 promoter-driven reporter activity in HCT8 cells, while the silencing of EN2 had the opposite results (Fig. 4j, k). Next, we wondered whether the EN2-CCL20 axis is clinically relevant. The results of IHC analysis showed that EN2 protein expression was correlated positively with the level of CCL20 (Fig. 4l). A positive correlation between EN2 and CCL20 mRNA expression was verified in GSE9348 and TCGA databases (Fig. 4m). The AUC of EN2 and EN2 combined with CCL20 were 0.733 and 0.895. This analysis indicated that EN2 combined with CCL20 for the CRC diagnosis was better than EN2 alone (Fig. 4n). Taken together, our results indicated that CCL20 was a downstream target of EN2 in CRC cells.

### EN2 promotes CRC proliferation and migration via upregulating CCL20

**Fig. 5:**
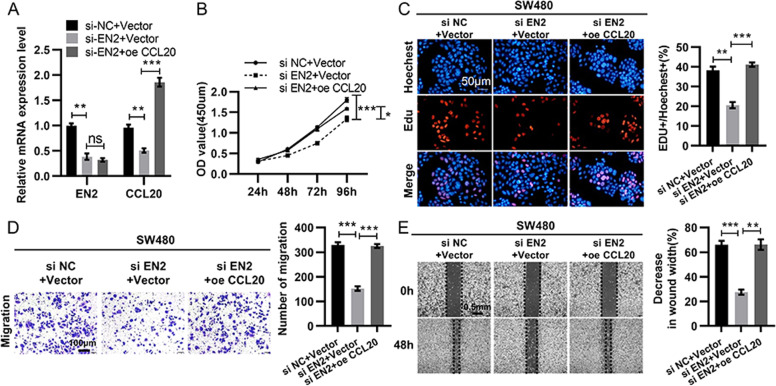
EN2 promotes cell proliferation and migration of CRC via activating CCL20 signaling. **a** The mRNA level of EN2 in SW480 cells transfected with vector, si-EN2 and si-EN2 + CCL20. **b**–**e** Cell proliferation and migration detected through CCK8 assay (**b**), Edu assay (**c**), transwell assay (**d**), and wound healing test (**e**) of SW480 cells transfected with vector, si-EN2, or si-EN2 + CCL20, respectively. **b**, **c** The capacity of cell proliferation determined by CCK8 assay (**b**) and cell clone-formation assay (**c**). **d** The capacity of cell migration assessed by transwell assay. Scale bar: 100 µm. **e** The capacity of cell migration assessed by wound healing test. Scale bar: 0.5 mm. Data are presented as mean ± SEM from three independent experiments. *P < 0.05, **P < 0.01, ***P < 0.001.

Since CCL20 acted as a direct target gene of EN2, we then performed functional recovery experiments to determine whether EN2 exerts functions by regulating CCL20 expression in CRC (Fig. 5a). SW480 cells with EN2 knocked down were transfected with OE-CCL20 and then the cell proliferation and migration abilities were measured. The results showed that the restrained proliferation and migration caused by the knockdown of EN2 could be reversed when CCL20 was upregulated (Fig. 5b–e). These data confirmed that EN2 promoted CRC proliferation and migration by regulating CCL20 expression.

## Discussion

Recent studies have shown that homeobox-containing genes, including HOX, EMX, PAX, EN, and so on, as key regulators contribute to the development and progression of a variety of human cancers^3,18,19^. As the human engrailed homolog, EN2 has been reported to correlate closely with human cancers such as prostate, breast, bladder, ovarian cancer, and non -small cell lung cancer^6–8,20,21^. However, it remains unclear whether EN2 has any role in colorectal cancer. Our study found EN2 expression level was higher in CRC tissues than that in tumor adjacent tissues on the basis of GES9478, TCGA, and our CRC specimens. The high expression of EN2 is significantly correlated with tumor size, histological grade, advanced TNM stage as well as poor survival of patients. We also found that EN2 knockdown could suppress CRC cell proliferation and migration in vitro and in vivo. These results indicate that EN2 may be an independent diagnostic and prognostic marker for CRC patients.

Pre-existing research had revealed that EN2 was located in the cytoplasm of breast and prostate cancer^7,17^. In this study, we showed that EN2 was mainly located in CRC cell nucleus, which means that EN2 act as a transcription factor to activate the expression of downstream genes in CRC. We further investigated the potential mechanism of EN2 in tumorigenesis of CRC by RNA sequencing, which revealed that EN2 might regulate wound healing, growth factor activity, cytokine activity, and TNF pathway, confirming that EN2 was crucial in CRC tumorigenesis. Next, we used the ChIP and luciferase assay to explore a direct binding site of the EN2 on the promoter region of CCL20, which provides evidence that CCL20 is regulated by EN2 transcription. Furthermore, the positive correlation between CCL20 and EN2 was verified in both mRNA and protein levels in CRC tissues.

CCL20 functions as a sort of chemokine existing in a variety of human tissues^8,22^. Also, CCL20 is highly expressed in colorectal cancer cells and contributes to cancer progression^23,24^. For example, in CRC, Yu X et al. and Cheng et al. demonstrated that CCL20 overexpression could promote cell proliferation and migration in CRC^25^. Additionally, Wang D et al. reported that the high level of CCL20 was closely associated with the poor survival of CRC patients. CRC cell-secreted CCL20 can recruit Tregs that further enhanced the chemoresistance of CRC cells to 5-FU^26^. Besides, CCL20 could influence the microenvironment via B and T cells, thus affecting CRC progression^27^. In our study, we discovered that EN2 was able to positively regulate the expression of CCL20 in CRC. Moreover, the restrained cell proliferation and migration caused by knockdown of EN2 could be reversed by upregulating CCL20. These results indicate EN2 exerts its function, at least in part, through regulating CCL20 expression.

In conclusion, our study indicates that EN2 is frequently upregulated in CRC tissues and associated with poor prognosis and thus can be a promising diagnostic and prognostic marker for CRC patients. In mechanism, EN2 promotes CRC cell proliferation and migration, at least partially, by regulating CCL20 expression. Taken together, these findings suggest that EN2 is an oncogene in CRC and may serve as a promising target for CRC prevention and therapy.

## Supplementary information

Supplementary Figure 1

Supplementary Figure legend
