# Supplementary figures and images for: EN2 as an oncogene promotes tumor progression via regulating CCL20 in colorectal cancer
Source: Cell Death Dis. 2020 Jul 30;11(7):604. doi: 10.1038/s41419-020-02804-3 (PMC7393501; doi:10.1038/s41419-020-02804-3)

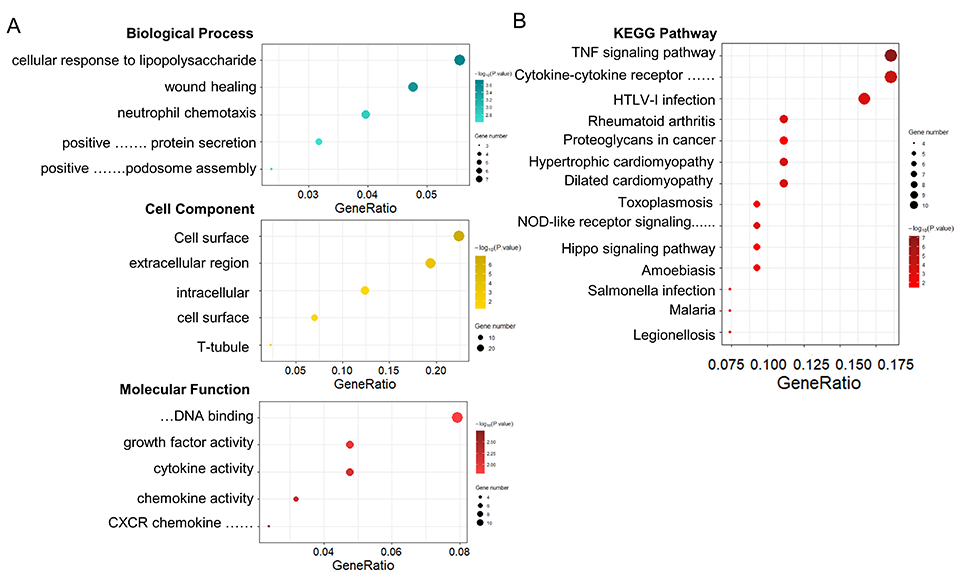

Supplement: Supplementary file 1 — Supplementary Figure 1 [file 41419_2020_2804_MOESM1_ESM.tif]
