# Supplementary material for: EN2 as an oncogene promotes tumor progression via regulating CCL20 in colorectal cancer
Source: Cell Death Dis. 2020 Jul 30;11(7):604. doi: 10.1038/s41419-020-02804-3 (PMC7393501; doi:10.1038/s41419-020-02804-3)
Supplement: Supplementary file 2 — Supplementary Figure legend [file 41419_2020_2804_MOESM2_ESM.docx]

Supplementary Figure 1: The results of RNAseq reveal potential downstream signaling of EN2

A-B) Gene Ontology terms (A) and KEGG pathway enrichment (B) of differentially expressed genes after EN2 was knocked down.

Table 1. Correlations between EN2 expression and clinicopathologic features in 165 colorectal cancer patients

The bold number represents the *P*-values with significant differences.
